# EXPLORING THE POWER OF DOCUMENTARY FILM TO CHALLENGE DEMENTIA-RELATED STIGMA AND SUPPORT DANCE FOR LIFE ENRICHMENT

**DOI:** 10.1093/geroni/igae098.2358

**Published:** 2024-12-31

**Authors:** Pia Kontos, Romeo Colobong, Jon Parr Vijinski, Devin Sodums, Rosanne Aleong, Rachel Bar

**Affiliations:** KITE Research Institute, Toronto Rehabilitation Institute – University Health Network; Dalla Lana School of Public Health, University of Toronto, Toronto, Ontario, Canada; KITE Research Institute, Toronto Rehabilitation Institute – University Health Network, Toronto, Ontario, Canada; Rotman Research Institute, Baycrest Academy for Research and Education, Toronto, Ontario, Canada; Rotman Research Institute, Baycrest Academy for Research and Education, Toronto, Ontario, Canada; Rotman Research Institute, Baycrest Academy for Research and Education, Toronto, Ontario, Canada; Canada’s National Ballet School, Toronto, Ontario, Canada

## Abstract

Dance has been shown to support empowerment, sociability, and creative self-expression; however, it is rarely adopted with the explicit intention to support these aspects of engagement in the context of dementia care. Instead, dance is primarily adopted as a form of therapy to improve cognitive and physical health outcomes. This is far too narrow given there is research that demonstrates the power of dance to increase social inclusion by supporting embodied self-expression, creativity, and social engagement of people living with dementia. In order to foster broad community awareness building and education about the value of dance for life enrichment and the importance of supporting this in dementia care settings, we developed a short documentary film – Dancer Not Dementia. To evaluate the impact of the film, we conducted focus groups and/or interviews with people living with dementia, family carers, government policy makers, formal care providers affiliated with long-term and community-based dementia care, members of the dance community, and the general public. Data were collected within one week of watching the film and then again 8-12 weeks later. Our analysis illustrates the effectiveness of Dancer Not Dementia in reducing stigma by demonstrating changes in understandings of dementia and changes in practice. Our analysis also includes attention to how the film, as a form of cultural production, deepened engagement and facilitated transformation. This research contributes to a growing understanding of how the arts may challenge entrenched and oppressive attitudes and social relations, and support more inclusive and relational approaches to caring.

